# The contingent effects of supervisor positive gossip on employee receivers: the moderating role of performance goal orientation

**DOI:** 10.3389/fpsyg.2025.1516309

**Published:** 2025-08-19

**Authors:** Fangliang Zhang, Chunling Zhu

**Affiliations:** ^1^School of Business, Guangxi University, Nanning, China; ^2^Renmin Business School, Renmin University of China, Beijing, China

**Keywords:** supervisor positive gossip, self-efficacy, performance goal orientation, social comparison, workplace gossip

## Abstract

While research on supervisor gossip has sought to provide a balanced examination of its potential benefits and drawbacks for employees, there remains a significant disparity in the attention given to positive versus negative gossip. By integrating social comparison theory and goal orientation theory, we propose that the impact of supervisor positive gossip on employee receivers’ self-efficacy and job performance is contingent upon the level of employees’ performance goal orientation (PGO). We argue that high-PGO employees are expected to experience lower levels of self-efficacy upon receiving supervisor positive gossip, whereas low-PGO employees are anticipated to experience higher self-efficacy. Additionally, we suggest that employees’ self-efficacy mediates the relationship between supervisor positive gossip and job performance. Dyadic data collected from 161 supervisors and 556 employees in a Chinese company support our hypotheses. Our findings contribute to a more nuanced discourse on the role of supervisor positive gossip in organizational dynamics and its implications for employee well-being and productivity.

## 1 Introduction

Supervisors often participate in *downward gossip*, which is defined as the supervisor’s informal and evaluative talk to an employee (i.e., the gossip receiver) about another employee who is not present (i.e., the gossip target) (Brady et al., 2017; Kuo et al., 2018; Bai et al., 2020; Zhu et al., 2023). Such type of gossip is considered more credible and influential than that initiated by employees, as it typically includes compelling narratives about job performance that are not readily available from any other sources (Kuo et al., 2018; Bai et al., 2020). Extant research has primarily focused on exploring the consequences of supervisor gossip on receivers, suggesting that supervisor negative gossip can undermine the leader-member exchange relationship (Kuo et al., 2018), while promoting receivers’ vicarious learning of norms and enhancing receiver performance (Bai et al., 2020).

However, research on the consequences of supervisor gossip suffers from two limitations. First, extant research has differentiated between the positive and negative gossip based on the valence of evaluations being made (Kurland and Pelled, 2000; Ellwardt et al., 2012), but with a general assumption that gossip tends to contain more negative connotations than positive ones (Kong, 2018; Wu L. Z. et al., 2018; Wu X. et al., 2018). This assumption may be biased, as evidence indicates that positive gossip and negative gossip are equally distributed within organizations (Spoelma and Hetrick, 2021). Supervisors might strategically use both types of gossip to reinforce norms and regulate deviant behaviors (Dores Cruz et al., 2021b; Zhu et al., 2022). Second, while the effects of negative gossip on work behavior can be either beneficial or detrimental (Kuo et al., 2018; Bai et al., 2020; Zhu et al., 2022, 2023), research on positive gossip has been notably limited to its favorable outcomes, such as its value for receivers’ self-improvement (Martinescu et al., 2014). Such view is generally predicated on the assumption of a homogenous receiver group, overlooking the fact that receivers possess diverse dispositional characteristics and may interpret positive gossip differently.

To address this concern, we pose the question: **D*oes supervisor positive gossip consistently *yield favorable outcomes for* employee receivers*? *Alternatively, could *it potentially lead to unintended negative consequences for some employee receivers?** Based on goal orientation theory (Dweck, 1986), we propose that the impact of supervisor positive gossip on receivers is dependent on the level of gossip receivers’ performance goal orientation (PGO). PGO describes an individual’s desire to affirm and establish their competence through outperforming their peers (Dweck, 1986; Elliot and Church, 1997). This orientation significantly influences how individuals interpret and respond to evaluative information within high-achievement contexts, such as the workplace (Lee et al., 2003; Poortvliet and Darnon, 2010; Downes et al., 2021).

In line with the social comparison perspective on gossip, gossip is seen as a conduit for conveying credible evaluative information that allows employee receivers to engage in social comparisons with the gossip targets (Wert and Salovey, 2004; Martinescu et al., 2014). Notably, when employees receive positive gossip from supervisors about their peers, they are likely to perceive these peers as superior performers and engage in *upward social comparisons* (Suls, 1977). These comparisons serve as a mirror for receivers to assess their own competence, which subsequently influences their performance (Blanton et al., 1999; Downes et al., 2021). We use the concept of self-efficacy to represent the outcomes of these self-assessments by employees. Self-efficacy pertains to an individual’s judgments of their capabilities to mobilize and apply skills necessary to accomplish specific performance objectives (Bandura, 1986).

**Figure 1:**
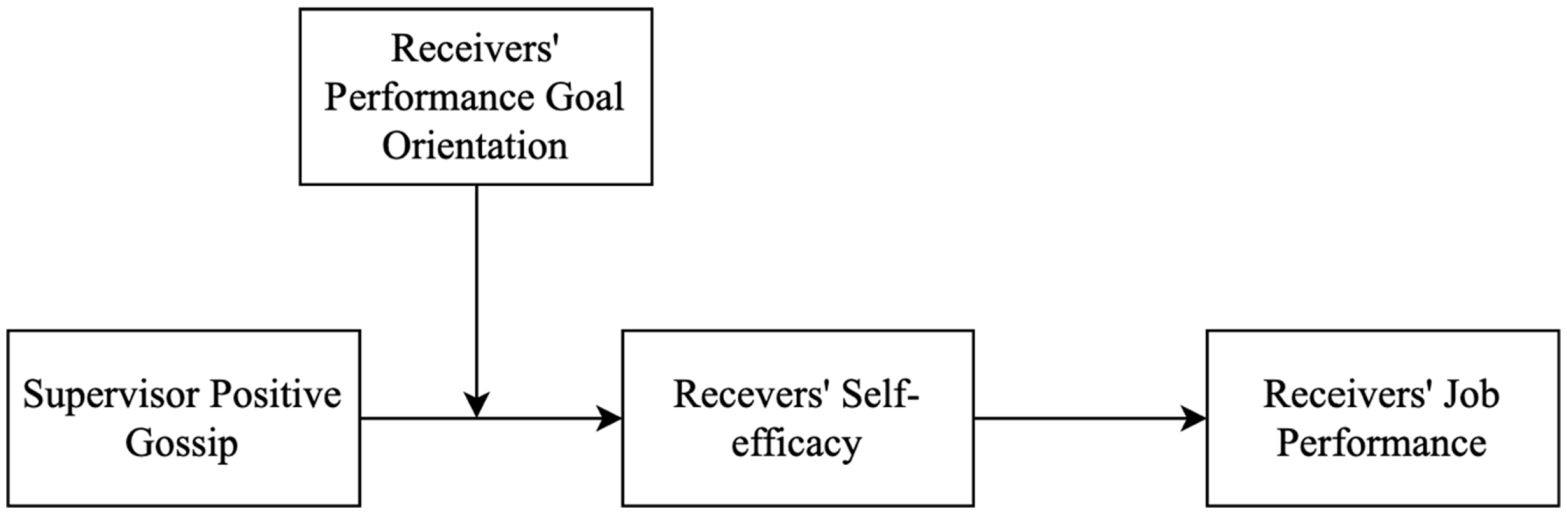
The theoretical model.

Based on goal orientation theory (Dweck, 1986), we contend that individuals with high PGO are more inclined to *contrast* themselves against gossip targets. Upon such comparisons, these high-PGO individuals might view themselves as less effective than the gossip targets, particularly when they are on the receiving end of supervisor positive gossip (Buunk and Gibbons, 2007). This realization can undermine their self-efficacy and job performance. In contrast, we expect that low-PGO receivers will exhibit an *assimilation* pattern. They are likely to view the targets of supervisor positive gossip as exemplary models to emulate, which in turn boosts their self-efficacy and drives them to pursue higher performance (Downes et al., 2021). The overall theoretical framework is illustrated in Figure 1.

Our research contributes to research on supervisor gossip, social comparison and goal orientation literature. First, by examining the contingent effects of supervisor positive gossip on employee receivers’ self-efficacy and job performance, our research provides a more nuanced understanding of when supervisor positive gossip may have unintended negative effects. While prior studies have highlighted gossip’s motivational or informative functions (Kuo et al., 2018; Bai et al., 2020; Zhu et al., 2023), we show that high-PGO employees may suffer reduced self-efficacy due to contrastive social comparisons. Second, our work offers cross-disciplinary insights by linking PGO from goal orientation theory to the social comparison function of gossip. Specifically, we argue that PGO functions as a proxy for individuals’ perceived attainability in upward comparisons, which determines whether gossip leads to assimilation or contrast effects (Lockwood and Kunda, 1997; Mussweiler et al., 2004). This perspective advances ongoing debates in social comparison research and highlights the psychological mechanisms that drive differential reactions to the same comparative information. Third, our study deepens the integration of social comparison theory into workplace gossip studies. By revealing the facilitative role of supervisor gossip in prompting receivers’ social comparisons, we reinforce the notion that “social comparison is deeply embedded in the fabric of organizational life” (Greenberg et al., 2007, p. 23). Supervisor gossip is not merely informal talk but a powerful social signal that employees interpret through the lens of their performance goal orientation, shaping their perceived self-worth and job performance.

## 2 Theory and hypotheses

### 2.1 Supervisor job-related gossip

**Figure 2:**
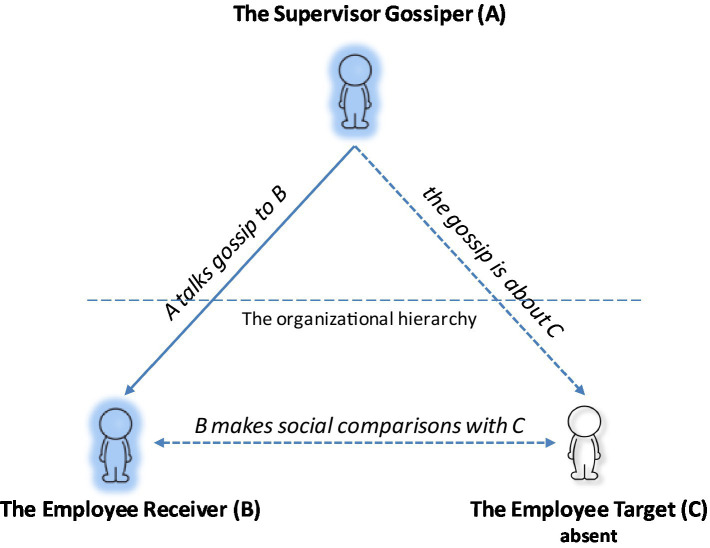
The gossip triad of supervisor gossip.

Recent advances in organization research acknowledge gossip as an intrinsic workplace activity that can hardly be eliminated but can be effectively managed (Grosser et al., 2012). Echoing the re-conceptualization of workplace gossip by Brady et al. (2017), we define *supervisor gossip* as informal and evaluative discourse from a supervisor to an employee about another employee who is not present. A typical instance of supervisor gossip involves a triad structure comprising a gossiper, a gossip receiver and a gossip target (Brady et al., 2017; Dores Cruz et al., 2021a). The gossip triad involved in supervisor gossip is illustrated in Figure 2. Compared to formal communication channels such as formal meetings and written documents, informal channels like gossip allow supervisors to convey messages and expectations more efficiently (Su et al., 2009; Bai et al., 2020; Zhu et al., 2023).

In the current study, we have chosen to focus specifically on *job-related* gossip, which pertains to discussions about employees’ work behaviors, including their job performance, diligence, credibility, work skills, and job morality (Kuo et al., 2015). This type of gossip is particularly relevant to receivers in high-achievement contexts like the workplace (Martinescu et al., 2014; Kuo et al., 2015). Our research concentrates on what we define as *supervisor job-related positive gossip,* which involves a supervisor sharing positive evaluations about an absent employee’s work behavior with another employee, such as compliments on the absent employee’s excellent job performance or their diligence (Ellwardt et al., 2012).

### 2.2 Supervisor positive gossip and employee receivers’ self-efficacy

Social comparison theory posits that individuals have a fundamental need to evaluate their own abilities. When clear benchmarks are lacking, individuals tend to compare themselves with peers as a means to gauge the relative standing of their abilities (Festinger, 1954). In the workplace, while some tasks have clear performance metrics, the true value of many work contributions often remains ambiguous. Consequently, given performance levels take on richer meaning through social comparisons (Downes et al., 2021). As a result, employees are continually in search of evaluative information about their peers to evaluate their own capabilities (Sedikides and Skowronski, 2000; Sedikides and Gregg, 2008; Martinescu et al., 2014). Research has shown that workplace gossip fulfills this social comparison need by providing evaluative information about coworkers in a manner that feels less confrontational than direct inquiries (Suls, 1977; Wert and Salovey, 2004).

Supervisor positive gossip, which narrates tales of how other employees successfully accomplished job tasks, can trigger receivers’ upward comparisons with the gossip targets, who are perceived as higher performers (Collins, 1996; Lockwood and Kunda, 1997). Studies suggest that the social comparisons triggered by supervisor gossip underscore the perceived performance discrepancies between receivers and targets (Hogg, 2000; Bai et al., 2020), impacting receivers’ self-concepts such as self-esteem, self-worth, self-efficacy (Lockwood et al., 2002; e.g., Buunk and Gibbons, 2007; Martinescu et al., 2014). When the focus of self-concept construct is job- or task-specific, it becomes closely intertwined with self-efficacy (Gist and Mitchell, 1992; Downes et al., 2021). Self-efficacy, which refers to an individual’s assessment of their capabilities to mobilize skills and execute actions to achieve specific performance goals (Bandura, 1986), is particularly pertinent in the workplace context where employees constantly adjust self-evaluations based on social comparisons with their coworkers.

Social comparison theory further suggests that individuals may exhibit two distinct responses to social comparisons, namely *assimilation* and *contrast* (Suls et al., 2002; Mussweiler et al., 2004; e.g., Gerber et al., 2018). *Assimilation* occurs when receivers’ self-efficacy aligns with that of the gossip targets, adopting a more positive stance upon receiving supervisor positive gossip (i.e., believing that one could emulate the target’s improvements). On the other hand, *contrast* arises when receivers’ self-efficacy diverges from that of the gossip targets, leading to more negative perceptions after being exposed to supervisor positive gossip (i.e., feeling inferior to the target) (Gerber et al., 2018; Matta and Van Dyne, 2020). These divergent reactions demonstrate that the effects of supervisor positive gossip on receivers’ self-efficacy can swing between positive and negative outcomes (Mussweiler et al., 2004). Therefore, we first propose a baseline hypothesis regarding the relationship between supervisor positive gossip and receivers’ self-efficacy. We will delve into the positive and negative nuances of this relationship in subsequent hypotheses, exploring the conditions under which each type of response is more likely to occur.

*Baseline Hypothesis: Supervisor positive gossip is related to employee receivers’ self-efficacy, and this relationship can be either positive or negative.*

### 2.3 The moderation of receivers’ PGO on the relationship between supervisor positive gossip and self-efficacy

Researchers have identified various factors that act as the “toggle switch” which influences whether social comparisons lead to assimilation or contrast effects. For example, when comparers feel psychologically close to the comparison targets (e.g., Lockwood and Kunda, 1997; Tesser, 1988) or share similar characteristics with the targets (e.g., Brown et al., 1992), they are more inclined to assimilate their self-evaluations towards the targets. The contrast effects occur under opposing conditions. Essentially, these moderators hinge on the comparers’ perceived attainability – the belief of whether they could realistically achieve similar outcomes as the targets in the future (Mussweiler et al., 2004).

In high-achievement contexts like the workplace, employees’ perceptions of their abilities significantly shape their expectations for their future performance (Payne et al., 2007). Those who view their competence as a fixed attribute may doubt their capacity for improvement through effort and tend to adopt performance goals as a result (Dweck, 1986). These individuals define competence in terms of outperforming others and are especially attuned to interpersonal comparisons (VandeWalle et al., 1999; Elliot, 2005). When exposed to upward comparisons, such as hearing praise about high-performing coworkers, they may experience threat, frustration, or inferiority (Elliot, 2005; VandeWalle et al., 1999). Accordingly, we propose that receivers’ PGO is a critical moderator that shapes whether supervisor positive gossip enhances or undermines receivers’ self-efficacy. We argue that PGO shapes how employee receivers interpret the supervisor positive gossip, either as an opportunity or threat, by influencing their perception of attainability. Although our study does not directly measure perceived attainability, we conceptualized PGO as a proxy for this belief: individuals high in PGO are more likely to interpret coworkers’ superior performance as unattainable, while those low in PGO are more likely to believe such outcomes are within reach.

Specifically, individuals with high PGO often engage in contrast comparisons, viewing others as competitors and themselves as adversaries (Dweck, 1986; Elliot and Church, 1997). For these individuals, positive gossip from supervisors about other employees can lead to the perception that others are outperforming them, triggering feelings of inadequacy and self-doubt (Buunk and Gibbons, 2007), particularly when they perceive the positively gossiped targets’ performance as unattainable. This perception reduces their self-efficacy, the belief that they are capable of achieving similar success, even if their motivation to perform remains high. Previous studies have indicated that receivers with high PGO, when making upward comparisons, frequently experience negative emotions such as self-directed frustration, resentment and envy towards high performers (Lockwood and Kunda, 1997; Smith, 2000), and they may fail to benefit from vicarious learning opportunities (Downes et al., 2021). Therefore, we expect that for high-PGO receivers, supervisor positive gossip may inadvertently undermine their confidence in bridging the perceived performance gap (i.e., self-efficacy) (Collins, 1996; Cohen-Charash and Mueller, 2007).

In contrast, employees with low PGO do not define their self-worth through outperforming others and are less likely to perceive coworker success as threatening. While they may not be high in learning goal orientation, their lower concern with perceived performance gap enables them to see supervisor positive gossip as useful insights rather than competition (Weber and Hertel, 2007; Downes et al., 2021). By learning from the behaviors and strategies contained in supervisor positive gossip, they can experience various mastery (Bandura, 1977), which enhances their belief in their own ability to achieve similar outcomes. These employees are more likely to interpret high performers as role models and view their success as attainable, thereby increasing their self-efficacy (Bandura, 1977; Lockwood and Kunda, 2000). This perspective aligns with Lockwood and Kunda’s (1997) finding that individuals can be inspired by high-performing “superstars” when they believe success is within their reach.

In sum, we argue that PGO determines whether supervisor positive gossip is interpreted through the lens of threat or inspiration, and thereby whether it decreases or increases self-efficacy. The rationale leads to our first hypothesis:

*Hypothesis 1: Receivers’ PGO moderates the relationship between supervisor positive gossip and receivers’ self-efficacy such that the relationship is negative for high-PGO receivers and positive for low-PGO receivers.*

### 2.4 The mediation of self-efficacy between supervisor positive gossip and job performance

We further anticipate that receivers’ self-efficacy serves as a mediator in the relationship between supervisor positive gossip and receivers’ job performance. Self-efficacy, which signifies an individual’s assessment of their capabilities to execute courses of action necessary for dealing with prospective situations (Bandura, 1982), fosters both the ambition to pursue higher goals and the persistence to overcome challenges and obstacles (Bandura, 1997). Meta-analyses have confirmed a robust positive link between self-efficacy and work-related performance (Stajkovic and Luthans, 1998).

Individuals with high self-efficacy are more inclined to set lofty performance goals and exhibit a stronger commitment to achieving them, leading to superior performance levels (Downes et al., 2021). In contrast, those with lower self-efficacy often harbor modest aspirations and lower performance expectations, potentially abandoning their efforts early and failing to accomplish their tasks (Bandura, 1977, 1986). Taken together, the assertion that receivers’ self-efficacy is positively related to job performance can be integrated with the notion that supervisor positive gossip influences self-efficacy. This leads to the proposal that self-efficacy acts as a mediator in the relationship between supervisor positive gossip and job performance. We thus propose the following hypothesis:

*Hypothesis 2: Receivers’ self-efficacy mediates the relationship between supervisor positive gossip and receivers’ job performance.*

### 2.5 An integrative moderated mediation model

Thus far, we have developed a theoretical framework for the first-stage moderation effect of receivers’ PGO (Baseline Hypothesis and Hypothesis 1), and the mediation of receivers’ self-efficacy between supervisor positive gossip and receivers’ job performance (Hypothesis 2). The theoretical rationales behind these hypotheses altogether suggest an integrative first-stage moderated mediation model, which indicates receivers’ PGO moderates the indirect effect of supervisor positive gossip on receivers’ performance via receivers’ self-efficacy. Accordingly, we propose the following hypothesis.

*Hypothesis 3: Receiver’s PGO moderates the indirect effect of supervisor positive gossip on receivers’ job performance via self-efficacy, such that the indirect effect is negative for high-PGO receivers, and positive for low-PGO receivers.*

## 3 Method

### 3.1 Research context

Our multi-wave and multi-source data were collected from a nation-wide retailer company in China, which operates over 500 branch stores across central mainland China. The company is dedicated to supplying fresh dairy products to local customers. We communicated the purpose and significance of our research with the management team, and gained their support to conduct the questionnaire survey across its branch stores. For each branch store, there are one manager (i.e., the supervisor) and several salespeople (i.e., the employees). During the workday, the employees are assigned with different tasks and have separate working areas. For example, the employee responsible for check-out service works behind the counter, while the employee that serves customers normally works near the store entrance. The manager, meanwhile, walks around the store inspecting their work. Moreover, the employees take turns to have their mealtime. In other words, the setting of separate work areas and staggered mealtime makes it possible for the managers to talk gossip to an employee about an absent employee. Besides, the managers and employees are all highly aware of the significance of their work to the store’s overall business performance because their salary is closely linked to the store’s sales revenue. Therefore, we deem this company an optimal setting for studying supervisor positive gossip and its implications for employee job performance, given the prevalent opportunities for such interactions and the high value placed on job performance.

### 3.2 Participants and procedures

We conducted a three-wave data collection with the intention to reduce the likelihood of common method bias (Podsakoff et al., 2003). We explained to all participants (both supervisors and their employees) that the purpose of this study was to examine the effectiveness of current human resource practices and to seek out the possible directions for improvements. Separate questionnaires were administered to employees and supervisors. Participants were informed of the details of the study, the voluntary nature of participation and the assurance of anonymity before they filled out the questionnaire. Each employee was assigned a personal code to fill out the questionnaire so we can match their responses with their supervisors’. We collected data with a one-month interval. In Phase 1 (P1), we asked employees to provide their demographic information, and rate their perceptions of the supervisor gossip they received in the past month, and their PGO. In Phase 2 (P2), which was conducted one month after P1, the employees reported their perceived self-efficacy in the past month. In Phase 3 (P3), which was conducted one month after P2, the employees’ direct supervisors were asked to evaluate employees’ job performance in the past month.

We received a list of 719 randomly selected employees and their direct supervisors. 676 of them submitted their questionnaires in P1. In P2, we received 658 usable employee questionnaires. Finally, in P3, questionnaires were distributed to the employees’ direct supervisors. After matching, we obtained a final sample of 556 complete supervisor-employee dyads with a response rate of 77.33%, which constituted the basis for our analysis. Taken together, the final sample used for this study consisted of 161 supervisors and their 556 employees. Of the 556 employees, 73.38% are female. Their average age is 32.5, and the average tenure is 3.79 years. The average time spent under their supervisor’s supervision is 2.10 years, and 39.03% of employees’ education level is junior college and above. Of the 161 supervisors, 66.46% are male. The average age is 31.44, and the average tenure is 3.86 years. 62.11% of supervisors’ education level is junior college and above.

### 3.3 Measures

As the survey was conducted in China, we used Chinese as our survey language. Nonetheless, all measures in this study were originally developed in English. To ensure the equivalency of meaning and minimize the misunderstanding, we followed the commonly-used back-translation procedures (Brislin, 1980). Specifically, the scales were first translated from English into Chinese by a researcher and then were back translated into English by another researcher. Both researchers have years of overseas study experience. Finally, a bilingual researcher compared the English and Chinese versions of these measures and made modifications to resolve the minor discrepancies. This rigorous process helped to ensure the accuracy and reliability of the survey measures in the context of our study.

#### 3.3.1 Supervisor positive gossip

We modified Kuo et al.’ (2015) five-item job-related gossip scale to measure supervisor positive gossip separately. The original scale was designed to evaluate an employee’s positive and negative gossip about his or her coworker, so we reworded the items to reflect the supervisor’s positive gossip about employees for the purpose of our study. A sample item of supervisor positive gossip is “have your supervisor recently talked to you about your coworker’s diligence and dedication to work?” All of the items were evaluated on a 5-point response scale (1 = never, 2 = seldom, 3 = sometimes, 4 = often, 5 = always). Cronbach’s alpha for supervisor positive gossip scales is 0.95.

#### 3.3.2 Self-efficacy

An eight-item scale developed by Chen et al. (2001) was used to measure receivers’ self-efficacy. The response options ranged from 1, “strongly disagree,” to 5, “strongly agree.” A sample item is “when facing difficult tasks, I am certain that I will accomplish them.” Cronbach’s alpha for this scale is 0.92.

#### 3.3.3 Performance goal orientation

An eight-item scale developed by Button et al. (1996) was used to measure PGO. The response options ranged from 1, “strongly disagree,” to 5, “strongly agree.” A sample item is “I feel smart when I can do something better than most other people.” Cronbach’s alpha for this scale is 0.95.

#### 3.3.4 Job performance

Given that employees’ contributions to store revenue are highly interdependent and not directly traceable to individual performance for all roles (For instance, some employees are responsible for checkout services or stocking inventory rather than direct sales), we argue that store-level sales cannot reliably capture each employee’s individual performance. Therefore, we used a six-item subscale developed by Tsui et al. (1997) to measure job performance. The response options ranged from 1, “strongly disagree,” to 5, “strongly agree.” Of the original 11 items, six assessed employees’ basic task performance in terms of task quantity, quality, and efficiency. The supervisor rated the extent to which he or she agreed with the items describing the employee’s performance as better than the average level. The sample items include “this employee’s quantity of work is higher than average” and “this employee strives for higher quality work than required.” Cronbach’s alpha for this scale is 0.93.

#### 3.3.5 Control variables

As negative gossip has been found to influence individuals’ self-evaluations and work behavior (Martinescu et al., 2014; Kong, 2018; e.g., Bai et al., 2020; Spoelma and Hetrick, 2021), we controlled for supervisor negative gossip using Kuo et al. (2015) 5-item negative gossip scale. A sample item is “have your supervisor recently talked to you about your coworker’s carelessness and poor work engagement?” Cronbach’s alpha for this scale is 0.85.

Since PGO is a paired variable with learning goal orientation (LGO), we also controlled for employees’ LGO using Button et al. (1996)‘s eight-item scale. A sample item is “the opportunity to learn new things is important to me.” Cronbach’s alpha for this scale is 0.82. Although there is sparse empirical evidence that women gossip more frequently than men (Foster, 2004; Grosser et al., 2012; Robbins and Karan, 2020), the content or valence of gossip among female and male differs (Levin and Arluke, 1985; Watson, 2012). In order to be consistent with prior gossip studies, we controlled for supervisors’ and employees’ biological sex. We also controlled for their age based on some evidence showing that younger women gossip more about rivals (Massar et al., 2012) and the elders who live alone gossip more about their acquaintances (Torres and Warren-Findlow, 2019). Besides, we controlled for supervisors’ and employees’ working tenure, education level and employees’ working time with the supervisor in consideration of their possible influences on job performance (Ng and Feldman, 2009, 2010).

### 3.4 Analytical strategy

Although all studied variables were conceptualized and measured at the individual level (Level 1), employees were nested within supervisors, introducing potential non-independence in the data. To assess this, we conducted null models without predictors and calculated the intraclass correlation coefficient [ICC(1)] for the outcome variable, employee job performance. The ICC(1) of employee job performance is 0.20, which exceeds the commonly accepted threshold of 0.10 (LeBreton and Senter, 2008) and indicates medium between-group variances. This justifies the use of multilevel modeling approaches.

Accordingly, we conducted the two-level analysis in Mplus 8 with the TYPE = TWOLEVEL command to account for the between-group variances (Muthén and Muthén, 2017). All focal predictors (e.g., supervisor positive gossip, PGO and self-efficacy) were modeled at the within-group level (%WITHIN%), while group-level control variables (i.e., supervisors’ age, gender, tenure and education) were modeled at the between-group level (%BETWEEN).

We used Monte Carlo integration with 10,000 iterations for indirect effect testing, and the significance of mediation and moderated mediation effects was evaluated using bias-corrected bootstrap confidence intervals (95%). Model fit was assessed using standard fit indices including CFI, TLI, RMSEA and SRMR.

## 4 Results

### 4.1 Descriptive statistics and confirmatory factor analysis

All variables’ means, standard deviations, reliability scores and correlations are reported in Table 1. We find significant correlations between supervisor positive gossip and receivers’ self-efficacy (*γ* = 0.24, *p* < 0.001), and between self-efficacy and job performance (γ = 0.16, *p* < 0.001), and between supervisor positive gossip and job performance (γ = 0.10, *p* < 0.05). These significant correlations warrant further investigations on their relationships.

**Table 1:** Descriptive statistics and correlations.

Variables	Mean	SD	1	2	3	4	5	6	7	8	9	10
Among group-level variables												
1. Supervisor sex	0.66	0.47										
2. Supervisor age	31.44	4.84	−0.17^*^									
3. Supervisor tenure (year)	3.86	3.69	−0.11	0.29^***^								
4. Supervisor education	1.74	0.66	0.26^***^	0.02	−0.11							
Among individual-level variables												
1. Supervisor positive gossip	2.80	1.17	**0.95**									
2. Supervisor negative gossip	1.22	0.50	0.12^**^	**0.85**								
3. Self-efficacy	4.12	0.58	0.24^***^	−0.06	**0.92**							
4. PGO	1.77	0.65	−0.33^***^	0.09^*^	−0.43^***^	**0.95**						
5. LGO	3.43	0.78	0.22^***^	−0.15^***^	0.15^***^	−0.23^***^	**0.82**					
6. Job performance	3.98	0.76	0.10^*^	−0.05	0.16^***^	−0.14^**^	0.05	**0.93**				
7. Employee sex	0.27	0.44	0.09^*^	0.09^*^	0.18^***^	−0.03	−0.00	−0.05				
8. Employee age	32.50	6.61	−0.04	−0.08^*^	0.01	−0.01	−0.02	0.12^**^	−0.31^***^			
9. Employee tenure (year)	3.79	3.79	−0.07	0.08	−0.02	0.03	−0.15^***^	0.08	−0.11^*^	0.28^***^		
10. Employee education	1.45	0.61	−0.01	0.08	0.07	−0.02	0.06	0.01	0.13^**^	−0.16^***^	−0.09^*^	
11. Time spent under supervision (year)	2.10	2.12	0.00	−0.05	0.02	−0.10^*^	−0.05	0.04	−0.10^*^	0.29^***^	0.42^***^	−0.13^**^

*N* = 556, Sex: “1”-male; “0”-female; Education: “1”-high school; “2”-junior college; “3”-bachelor’s degree; “4”-master’s degree or above; Tenure and time spent under supervision are measured in years. The bold values on the diagonal are Cronbach’s alphas. *p < 0.05, **p < 0.01, ***p < 0.001; Two-tailed tests.

We conducted a series of confirmatory factor analyses to evaluate the discriminant validity and goodness of fit of our hypothesized model. The *χ*^2^, comparative fit index (CFI), Tucker-Lewis index (TLI), root-mean-square error of approximation (RMSEA), and standardized root mean squared residual (SRMR) indicators were used to assess the fit of the hypothesized model (Bentler, 1990; Browne and Cudeck, 1993; Kline, 2005). As shown in Table 2, the hypothesized six-factor model yields better fit to the data (*χ*^2^*/df* = 3.40, CFI = 0.90, TLI = 0.90, RMSEA = 0.06, SRMR = 0.05) than the parsimonious models, and provides satisfactory fit to the data (Browne and Cudeck, 1993).

**Table 2:** Results of the confirmatory factor analysis.

Model	χ^2^	*df*	*χ^2^/df*	CFI	TLI	RMSEA	SRMR
1. 6-factor SPG, SNG, SE, PGO, LGO, PER	2463.94	725	3.40	0.90	0.90	0.07	0.05
2. 5-factor SPG + SE, SNG, PGO, LGO, PER	5173.17	730	7.09	0.74	0.72	0.11	0.13
3. 4-factor SPG + SE + LGO, SNG, PGO, PER	6482.99	734	8.83	0.66	0.64	0.12	0.15
4. 3-factor SPG + SE + LGO + PER, SNG, PGO	8901.25	737	12.08	0.52	0.49	0.14	0.18
5. 2-factor SPG + SE + LGO + PER, SNG + PGO	12759.83	739	17.27	0.29	0.25	0.17	0.23

SPG, supervisor positive gossip; SNG, supervisor negative gossip; SE, self-efficacy; PGO, performance goal orientation; LGO, learning goal orientation; PER, job performance; df, degrees of freedom; CFI, comparative fit index; TLI, Tucker–lewis index; RMSEA, root mean square error of approximation; SRMR, standardized root mean squared residual.

### 4.2 Hypotheses testing

We tested our hypotheses using two-level analysis in Mplus 8. The predictors of supervisor demographics are grand mean-centered at the group level, and the other predictors are group mean-centered (Enders and Tofighi, 2007). The baseline hypothesis posits a relationship between supervisor positive gossip and receivers’ self-efficacy. As shown in Model 2 of Table 2, supervisor positive gossip is positively associated with the receiver’s self-efficacy (*β* = 0.12; *p* < 0.001), which suggests that in general case, receivers would assimilate rather than contrast to the gossip targets of supervisor positive gossip.

**Figure 3:**
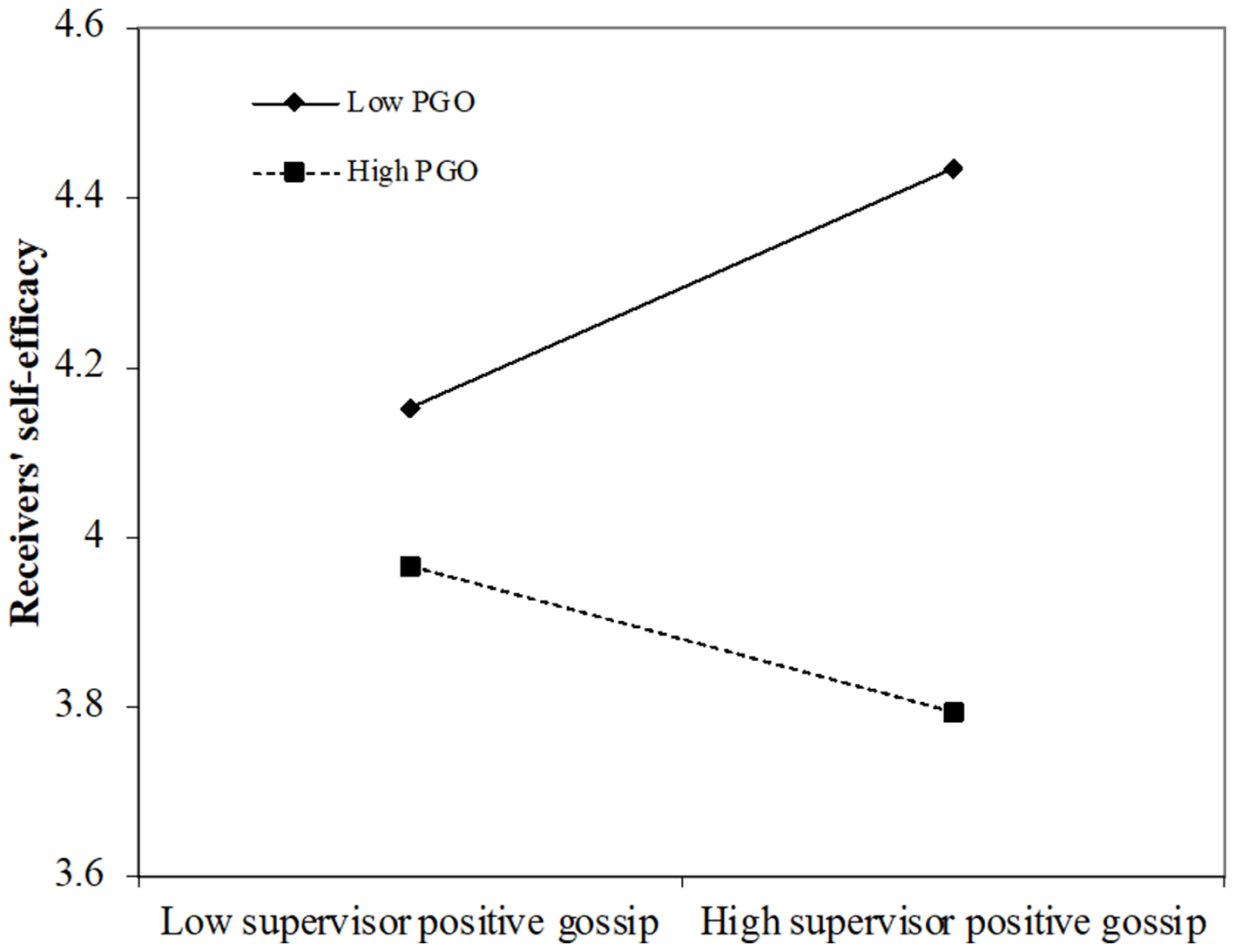
The interaction effect of supervisor positive gossip and pgo on self-efficacy.

Hypothesis 1 proposes the moderation effect of PGO. To examine this, we first form the interaction item by multiplying the group mean-centered variables of supervisor positive gossip with PGO to minimize multi-collinearity (Aiken and West, 1991). As shown in Model 3 of Table 3, the interaction is significantly related to self-efficacy (β = −0.23; *p* < 0.001). Therefore, we find preliminary support for Hypothesis 1. To specify the exact “toggle-switch” effect of PGO, we conduct simple slope tests at one standard deviation above and below the mean of PGO (Preacher et al., 2006), and the results demonstrate that the relationship between supervisor positive gossip and self-efficacy is significantly negative when PGO is high (β = −0.09; *p* < 0.05), and turns significantly positive when PGO is low (β = 0.15; *p* < 0.05), which confirms Hypothesis 1. We further illustrate the moderation effect in Figure 3 following the procedures proposed by Aiken and West (1991) and Dawson (2014).

**Table 3:** Results of two-level analysis.

Variables	Self-efficacy			Job performance		
	Model 1	Model 2	Model 3	Model 4	Model 5	Model6
Employee control variables						
Employee sex	0.18^**^	0.16^*^	0.17^**^	−0.16	−0.17^*^	−0.19^*^
Employee age	0.01	0.01	0.01	0.00	0.00	0.00
Employee education	0.09	0.09	0.07	0.01	0.02	0.00
Employee tenure	−0.00	0.00	0.00	0.02	0.02^*^	0.02^*^
Time spent under supervision	0.01	0.01	0.01	0.01	0.01	0.01
Supervisor negative gossip		−0.06	−0.01		−0.08	−0.07
Learning goal orientation (LGO)			0.04			
Supervisor control variables						
Supervisor sex	−0.03	−0.02	−0.03	0.08	0.08	0.08
Supervisor age	0.00	0.00	0.00	−0.01	−0.01	−0.01
Supervisor education	0.03	0.03	0.04	0.06	0.06	0.06
Supervisor tenure	0.01	0.01	0.01	−0.00	−0.00	−0.00
Independent variables						
Supervisor positive gossip		0.12^***^	0.03		0.06^*^	0.04
Mediator						
Self-efficacy						0.17^*^
Moderator						
Performance goal orientation (PGO)			−0.39^***^			
Interactions						
Supervisor positive gossip * PGO			−0.23^***^			
Supervisor negative gossip * PGO			0.14			
Supervisor positive gossip * LGO			0.00			
Supervisor negative gossip * LGO			0.15			

N = 556, ^*^p < 0.05, ^**^p < 0.01, ^***^p < 0.001. The predictors of supervisor demographics are grand mean-centered, and the other predictors are group mean-centered.

Hypothesis 2 states the mediation effect of self-efficacy. The results of two-level analysis indicate that supervisor positive gossip is positively related to self-efficacy (β = 0.12; *p* < 0.001, Model 2 of Table 3), and self-efficacy is positively related to job performance (β = 0.17; *p* < 0.05, Model 6 of Table 3). We ran the two-level analysis for the indirect relationship between supervisor positive gossip and job performance via self-efficacy, and results show that the unconditional indirect effect = 0.02 with the 95% CI = [0.003, 0.038], which supports Hypothesis 2.

Hypothesis 3 states that PGO moderates the indirect effect of supervisor positive gossip on receiver’s job performance through self-efficacy. To test the first-stage moderated mediation proposed by Hypothesis 3, we applied the moderated path analysis approach of Edwards and Lambert (2007) to estimate the indirect effects at one standard deviation above and below the mean of PGO levels. The results reported in Table 4 indicates that the conditional indirect effect between supervisor positive gossip and job performance via self-efficacy is significantly negative when PGO is high (Indirect effect = −0.02, 95% CI = [−0.031, −0.001]), and becomes significantly positive when PGO is low (Indirect effect = 0.03, 95% CI = [0.006, 0.046]). Moreover, the difference between the two conditional indirect effects is −0.04 with 95% CI = [−0.074, −0.010], which supports Hypothesis 3.

**Table 4:** Results of the moderated path analysis.

Moderator variable	Supervisor positive gossip (X) ➔ Self-efficacy (M) ➔Job performance (Y)				
	First stage	Second stage	Indirect effect	Indirect effect 95% confidence interval	
	P_MX_	P_YM_	P_MX_* P_YM_	Lower bound	Upper bound
Performance goal orientation					
High (mean+1 s.d.)	−0.09^*^	0.17^*^	−0.02^*^	−0.031	−0.001
Low (mean-1 s.d.)	0.15^*^	0.17^*^	0.03^*^	0.006	0.046
Difference	−0.24^*^	0.17^*^	−0.04^*^	−0.074	−0.010

Unstandardized path coefficients are reported. P_MX_ refers to path from supervisor positive gossip to self-efficacy, P_YM_ refers to path from self-efficacy to job performance, P_YX_ refers to path from supervisor’ positive gossip to job performance. ^*^p < 0.05, ^**^p < 0.01, ^***^p < 0.001. Two-tailed tests.

## 5 Discussion

The empirical examinations have indicated that the supervisor positive gossip’s influences on receivers’ self-efficacy and performance are contingent on receivers’ PGO levels. Specifically, for low-PGO receivers, supervisor positive gossip prompts them to view targets as exemplary figures, inspiring them to pursue comparable accomplishments. This, in turn, leads to an increase in their self-efficacy and performance. Conversely, for those with high PGO, a contrasting reaction occurs, upon hearing supervisor positive gossip, their self-efficacy and job performance are likely to decline. Overall, the level of PGO among receivers is a pivotal factor in determining whether supervisor positive gossip serves as a boon or a bane to their self-efficacy and job performance. This underscores the complexity of how supervisor gossip is perceived and internalized within an organizational context, with significant implications for workplace dynamics and individual motivation.

### 5.1 Theoretical contributions

Our findings contribute to the extant literature in the following ways. First, we contribute to workplace gossip research by extending the balanced view to positive gossip. While recent organizational research has recognized the dual nature of workplace gossip, highlighting both its benefits and costs for gossipers, receivers and targets (Brady et al., 2017; e.g., Bai et al., 2020; Tan et al., 2020; Sun et al., 2023; Zhong et al., 2023), much of this research has focused on negative gossip. Limited research has often assumed that positive gossip uniformly benefits receivers (Martinescu et al., 2014). We demonstrate that even positive gossip from supervisors can yield unintended negative consequences. Specifically, it lowers the self-efficacy of high-PGO employees who may interpret such upward comparisons as threats. Our findings underscore the importance of examining the complex and nuanced outcomes of positive gossip in the workplace.

Second, our research contributes to social comparison literature by strengthening its connections with supervisor gossip studies. Psychological scholars have long posited that gossip serves social comparison functions for all parties involved, providing an indirect and less confrontational means of satisfying individuals’ need for comparative information (Suls, 1977; Wert and Salovey, 2004). However, the integration of workplace gossip research with social comparison theory has been lacking in organizational literature, with most findings focusing on other functions of workplace gossip, such as reflective learning (Bai et al., 2020; Zhu et al., 2022), sense-making (Michelson et al., 2010; Mills, 2010), and social bonding (Beersma and Van Kleef, 2012; Dores Cruz et al., 2019). By investigating supervisor positive gossip as a vehicle for social comparison in the workplace, our study uncovers its potential to fulfill receivers’ social comparison needs. Supervisors’ formal authority over employees’ performance evaluations, compensation, and promotions lends credibility to their gossip, making it particularly salient to employees. By conceptualizing PGO as a proxy for perceived attainability, we extend the classic notion that comparison effects hinge on similarity and closeness (e.g., Brown et al., 1992; Lockwood and Kunda, 1997; Tesser, 1988), applying it in a new context of hierarchical gossip. Overall, this integration of social comparison approach with supervisor positive gossip enriches our understanding of workplace gossip functions and extends the applicability of social comparison theory cross disciplines.

Third, we contribute to goal orientation theory by showing that PGO not only predicts whether individuals engage in social comparisons but also shapes how they interpret and respond to the comparison information they receive. While researchers have established connections between goal orientation and social comparison theory, suggesting that individuals’ PGO prompts them to engage in social comparisons (Ames, 1992), few studies have examined how PGO shapes or determines individuals’ responses to these comparisons in high-achievement contexts (see exception: Downes et al., 2021; Zhu et al., 2023). Our study theorizes and finds that PGO *reverses* assimilation to a contrast path for high-PGO receivers. This finding underscores the value of considering PGO as a contingency factor that switches receivers’ reactions to supervisor positive gossip, and confirms the utility of goal orientation theory in clarifying the debate between assimilation and contrast effects in social comparison literature.

### 5.2 Practical implications

Our research offers several practical implications for supervisors, employees and organizations. First, recognizing that gossip is an inherent aspect of human social interaction, it is impractical for supervisors to eradicate it from the workplace entirely (Grosser et al., 2012; Dores Cruz et al., 2021a). However, its impact can be managed to some extent. Supervisors can strategically mitigate the adverse effects of gossip while leveraging its potential benefits. Our findings indicate that even positive gossip about employees can inadvertently harm individuals with high PGO. Therefore, we advise supervisors to utilize positive gossip judiciously as a motivational tool. Specifically, supervisors might consider engaging in positive gossip with low-PGO employees to inspire them to emulate high performers and set ambitious goals. Conversely, supervisors should be cautious about sharing positive gossip with high-PGO employees, as this may inadvertently diminish their self-efficacy for higher performance.

Second, for employees, being aware of the social comparison triggered by supervisor gossip and its potential effects is crucial. Since individuals can embrace both performance goals and learning goals simultaneously (Button et al., 1996), we suggest high-PGO employees temper their performance goals when exposed to supervisor positive gossip. Instead, they should adopt a constructive learning mindset towards the targets of supervisor positive gossip. Moreover, organizations should recognize that even well-intentioned comments by supervisors can be interpreted seriously by employees, leading to unintended consequences for work efficacy and performance. Organizations are encouraged to develop leadership coaching programs focused on communication skills and establish formal channels for performance feedback. In addition, we recommend that organizations adopt absolute performance evaluation standards rather than relative ones, particularly in settings where employees generally exhibit higher PGO. By relying more on formal feedback channels and absolute evaluation criteria, these organizations can mitigate the risk of employees experiencing reduced self-efficacy due to informal feedback, such as supervisor positive gossip.

## 6 Limitations and future directions

Despite its contributions, our research suffers from several limitations that warrant future investigation.

First, although our theoretical model draws on social comparison theory and posits perceived attainability as the key condition for whether upward comparisons yield assimilation or contrast effects (Mussweiler et al., 2004), we did not measure perceived attainability directly. Instead, we treated PGO as a proxy for individuals’ chronic beliefs about ability and the threat of upward comparisons. While theoretically grounded, this approach limits the precision of our inferences. Future research should consider directly assessing perceived attainability, possibly via experiments or validated measures, to better isolate its role in shaping responses to supervisor positive gossip.

Second, all data were collected from a single sales company in China, which may constrain the generalizability of our findings. Cultural norms surrounding authority, hierarchy, and indirect communication (such as gossip) may vary across cultures and industries, influencing how supervisor positive gossip is interpreted. We recommend future studies conduct cross-cultural and cross-industry replications to examine the robustness and boundary conditions of the observed effects.

Third, we used questionnaires to collect retrospective ratings of supervisor gossip, which lacks accuracy compared to real-time record methods such as laboratory experiments and naturalistic observations. Researchers have pointed out that since gossip is more like a private behavior, it is especially sensitive to research methods (Wert and Salovey, 2004). We acknowledge innovative attempts like the Electronically Activated Recorder (EAR) method adopted by Robbins and Karan (2020) to capture workplace gossip in real time. We encourage future studies to adopt such experimental or observational approaches in the natural workplace settings for more accurate insights.

Fourth, all variables in our study were collected through quantitative surveys, which may limit the depth of understanding of how employees interpret gossip and attribute meaning to it. Future studies could benefit from qualitative approaches such as interviews or open-ended diary studies to explore employees’ subjective experiences and sensemaking processes in response to supervisor positive gossip. Such methods may uncover additional boundary conditions or mediating mechanisms that are difficult to detect through standardized measurements.

Finally, while our outcome variable, job performance, was rated by supervisors, it still relied on subjective evaluations. Future research could incorporate objective indicators, such as actual sales figures or performance metrics, to more accurately assess the behavioral implications of gossip-related processes and mitigate potential biases in supervisory ratings.

## 7 Conclusion

By adopting a balanced view of workplace gossip, our research delves into the contingent effects of supervisor positive gossip on employees’ self-efficacy and job performance. Through the lens of social comparison theory and goal orientation theory, we found that supervisor positive gossip prompts receivers to make upward comparisons with the gossip targets, thereby enhancing their self-efficacy and job performance for low-PGO receivers, while diminishing the self-efficacy and job performance of high-PGO receivers. We aspire for this research to stimulate broad academic curiosity in the investigation of supervisor gossip, especially concerning the varied outcomes it may have on the individuals involved.

## Data Availability

The raw data supporting the conclusions of this article will be made available by the authors, without undue reservation.
